# Enhancement of Energy Metabolism in Skeletal Myocytes Protects Against Age‐Related Sarcopenia

**DOI:** 10.1111/jcmm.70588

**Published:** 2025-05-12

**Authors:** Andrey Y. Vinokurov, Pavel A. Bazhenov, Marina Y. Pogonyalova, Evgenia S. Seryogina, Ekaterina A. Vetrova, Larisa Andreeva, Andrey Y. Abramov, Plamena R. Angelova

**Affiliations:** ^1^ Cell Physiology and Pathology Laboratory Orel State University Orel Russia; ^2^ Mitocholine Ltd. London UK; ^3^ Department of Clinical and Movement Neurosciences UCL Queen Square Institute of Neurology London UK

**Keywords:** energy metabolism, glutathione, mitochondria, myotubes, reactive oxygen species, sarcopenia

## Abstract

Skeletal muscles constantly consume energy, and this consumption level increases correspondingly to the levels of physical activity. Mitochondrial energy metabolism requires constant supplementation with oxygen and substrates for ATP production. Limitation of the mitochondrial substrate supply leads to energy deprivation, which may be followed by sarcopenia and weight loss. Activation of mitochondrial energy metabolism can also stimulate the production of reactive oxygen species and oxidative stress. Here, we studied the effect of various mitochondrial substrates on the energy metabolism of primary skeletal myotubes and how it affects redox balance. We found that as individual components—glutamate, succinate, nicotinamide (NAM) as well as in combination—dicholine succinate (DISU) plus NAM, they increase mitochondrial membrane potential, alter NADH and FAD redox indices, which leads to an increased energy capacity of the skeletal myotubes. Changes in mitochondrial metabolism increased ROS production in mitochondria and cytosol but induced only a minor decrease in the level of the endogenous antioxidant reduced glutathione. Supplementation of young and aged rats with DISU + NAM through the drinking water for 7 days significantly increased myotube diameter in both age groups. Thus, provision of the myotubes with mitochondrial metabolism substrates activates energy metabolism and increases energy capacity but has no effect on oxidative stress. Moreover, it increases myotubes' diameters in young and aged rodent sarcopenia models in vivo.

## 1 Introduction

Muscles are the largest and most energy‐consuming organ of the human body. Skeletal muscles account for 40%–50% of the lean body mass [1, 2, 3]. Muscles play an important role in posture maintenance, exercise tolerance and temperature regulation. The high levels of substrate consumption as well as production and release of metabolites define muscles as a metabolism regulator [2, 4].

About 30% of the body's resting energy expenditure and almost 100% of the whole‐body energy consumption increases during physical activity consumed by skeletal muscles and heart [3, 5]. Among the most energy‐consuming processes that provide muscle contraction are the ion transport (mainly Ca^2+^‐transport into endo‐ and sarcoplasmic reticulum by SERCA and Na^+^/K^+^‐transport by ATPases) and the chemo‐mechanical transduction of the myosin–actin interaction (myofibrillar ATPase) [6, 7]. Intracellular steady‐state ATP concentration (5–8 mM) is sufficient for a few seconds of physical activity, and its depletion can be rapidly compensated by the creatine kinase reaction, which is the secondary ATP source, while the combination of glycolysis and oxidative phosphorylation (OXPHOS) primarily generates ATP by using intracellular glycogen or blood‐circulating glucose and glycolysis‐produced pyruvate, fatty acids or amino acids as substrates [4, 6, 8].

The balance between glycolysis and OXPHOS depends on the age, type and physiology of muscle fibers and cells as well as the type of physical activity [8, 9]. Although glycolysis is dominant in foetal muscle cells, the role of OXPHOS dramatically increases during the later periods [10]. As it was shown for artery smooth muscle cells, these two routes give approximately equal steady‐state ATP levels but differ in the reserve capacity—1.25 for glycolysis and 3.5 for OXPHOS [11]. Anaerobic glycolysis is the preferable bioenergetic pathway in fast‐twitch fibres with a higher fatigability, strength of contraction and lower oxidative capacity [12]. It should be noted that age‐related alterations lead to a significant decrease in the reserve capacity, especially in the case of (OXPHOS) [6].

Influencing the bioenergetics of muscle cells, mitochondria seem to be a very important component of cellular metabolism regulation. Thus, AMP‐activated protein kinase (AMPK), a key player in maintaining energy supply, impacts mitochondrial function, biogenesis, mitophagy and mitochondrial dynamics processes [13]. On the other hand, by producing reactive oxygen species (ROS) mitochondria can activate AMPK or increase insulin‐independent glucose consumption. Besides that, mitochondrial ROS elevation in the post‐exercise period leads to exercise adaptations. Proper mitochondrial function is an important condition for muscle regeneration because of an increase in energy requirement for myoblasts differentiation. The same reason determines the necessity of healthy mitochondria in the process of muscle stem cells activation, which can be delayed by inhibition of autophagy, including selective utilisation of mitochondria [14].

Muscle weakness and increased muscle atrophy due to bioenergetic disturbances are the signs of several diseases and metabolic disorders and can also be age‐associated. An impairment of the bioenergetic status, cell cycle disturbances and increased apoptosis were shown for myoblasts and myotubes of patients with congenital muscular dystrophy type 1A and Leigh syndrome [9]. Shift in the bioenergetic profile of smooth muscle cells may lead to de‐differentiation and increased proliferation of cells with subsequent rise of vascular diseases risk [11]. Skeletal muscle cells in patients with chronic fatigue syndrome are characterised by dysfunction in glucose oxidation [15]. Upregulation of glycolytic, apoptotic and hypoxic pathways with increased glucose, amino acids and fatty acids uptake, as well as downregulation of the pentose phosphate pathway and fatty acids oxidation, were shown in myotubes from donors with obesity compared to myotubes from lean donors [4]. The muscles of an elderly person are characterised by the higher level of fatigue compared to the young one due to a decreased ATP production and a greater increase in protons and inorganic phosphate concentration. Mitochondrial alterations leading to ROS overproduction are part of the mechanism of myofiber atrophy via degradative pathways during chronic muscle disuse [16]. Increased loss of muscle mass and function is the main sign of sarcopenia [17]. Predominantly affecting adults of older age, sarcopenia can be accelerated by genetic and lifestyle factors in mid‐age. It ranges from 5% to 50% depending on gender, age, pathological conditions and diagnostic criteria, and has a great personal, social and economic impact when untreated [18, 19].

Dietary supplementation seems to be one of the most preferable ways to prevent muscle atrophy in advanced age. It was shown that betaine could promote muscle fibres differentiation and increase myotubes size that leads to reduction of fatigue [20]. Glutamine supplementation demonstrates stimulation of satellite cell proliferation with subsequent differentiation to myoblasts and fusion with myofibers [21]. Dietary succinate increases endurance exercise ability, myosin heavy chain I expression, aerobic enzyme activity, oxygen consumption and mitochondrial biogenesis in mouse skeletal muscle that associates with remodelling of muscle fibres from fast‐ to slow‐twitch [22]. Several dietary strategies have been developed for sarcopenia‐affected patients. The most important components of them are essential amino acids, medium‐ or long‐chain triglycerides, carbohydrates, vitamins (especially vitamin D), l‐carnitine, creatine, minerals and fibres [19].

Previously, we have shown that supplementation of the neurons and astrocytes with mitochondrial substrates or the complex of dicholine succinate (DISU) plus nicotinamide (NAM) leads to enhanced energy production that allows for neuroprotection in Parkinson's disease cellular models [23]. Here, we studied if mitochondrial substrates are able to increase energy metabolism in skeletal myotubes and whether it induces oxidative stress. We have found that mitochondrial substrates and particularly the combination of DISU+NAM increase mitochondrial membrane potential and modify NADH and FAD redox indices. Further, it led to the increase of energy capacity in skeletal myotubes. Importantly, although mitochondrial substrates increased ROS production in the mitochondria and cytosol of myotubes, they did not induce oxidative stress and a substantial decrease of reduced glutathione (GSH). Supplementation of the DISU+NAM combination to drinking water for 7 days increased the diameter of the myotubes in mid‐age and aged rats as a model of sarcopenia.

## 2 Materials and Methods

### 2.1 Animals

For the experiments, male Wistar rats (1‐ and 1.5‐year‐old) and Wistar rat pups 2–4 days post‐partum were used. Adult rats were housed with free access to water and food under a 12:12 h light/dark photocycle in an air‐conditioned room at 22°C–24°C. All of the animal procedures were performed in compliance with the ARRIVE guidelines and approved by the Institutional ethical committee of Orel State University (No. 18 dated 21 February 2020) in compliance with Russian Federation legislation.

#### 2.1.1 Fibre Size Experiment

Animals were treated with DISU+NAM through the drinking water ad libitum at a concentration of 100 μM for 7 days. To analyse the diameter of muscle fibres, acute sections of transverse striated muscles of hind legs were incubated in saline solution in the presence of the fluorescent probe Fluo‐4 AM for 30 min.

Images of the fluo‐4 fluorescence in fibres were obtained with a Zeiss 900 LSM with an integrated META detection system using 488 nm laser for excitation. Measurement of the diameters of fibres was done using Zeiss software.

### 2.2 Primary Muscle Culture

To prepare mixed cultures of skeletal myocytes and fibroblast cells, hindlimbs of postnatal day 2–5 (P2‐5) rat pups were taken and skin and fat tissues were carefully removed [13]. The muscle tissues were then digested in 0.2% collagenase (Gibco, USA) solution at 37°C for 30–40 min. The reaction was stopped by adding 2–3 mL FBS (Biological Industries, Israel) and the tissue suspension was centrifuged at 2010 rpm for 10 min. The cell pellet was resuspended in pre‐warmed growth medium (DMEM containing 20% FBS, 100 IU/mL penicillin and 200 IU/mL streptomycin) (Gibco, USA) and plated on 22 mm coverslips pre‐coated with 0.2% gelatine (PanEco, Russia) for 1 h at 37°C. Cells will then be cultured in a humidified CO_2_ incubator (5% CO_2_ in air) at 37°C. After 2–3 days, the medium was changed to a low FBS containing medium (DMEM containing 10% FBS without antibiotics), and the medium was changed every 3–4 days. All experiments were performed between days in vitro 5–11. All experiments were conducted at least three times from three different animals.

### 2.3 Measurement of Mitochondrial Membrane Potential

The mitochondrial membrane potential (ΔΨm) was measured by loading cells with 25 nM tetramethylrhodamine methyl ester (TMRM) (Invitrogen by Thermo Fisher Scientific, USA) in a HEPES‐buffered HBSS (Gibco, USA) for 45 min at a room temperature. Z‐stack images were obtained by confocal microscopy (Zeiss LSM 900) (excitation 560 nm emission above 610 nm) and the maximum fluorescence intensity was used as a measure of ΔΨm. Additionally, 2 μM FCCP (Sigma‐Aldrich, USA) was used for the depolarisation of mitochondria and calibration of the signal. The difference in the TMRM signal before and after FCCP application was taken as Δψm in the single cells. Illumination intensity was kept to a minimum (at 0.1%–0.2% of laser output) to avoid phototoxicity, and the pinhole was set to give an optical slice of ~2 μm.

### 2.4 NADH and FAD Fluorescence Measurements

NADH autofluorescence was measured using an epifluorescence inverted microscope equipped with a ×40 fluorite objective. Excitation light at a wavelength of 340 nm was provided by a Xenon arc lamp, the beam passing through a monochromator (Cairn Research, UK). Emitted fluorescence light was reflected through a 455‐nm long‐pass filter to a cooled CMOS camera (Teledyne Photometrics, UK). Imaging data were collected and analysed using MetaFluor imaging software (Molecular Devices, UK).

FAD fluorescence was measured using Zeiss LSM 900 with excitation at 488 nm and emission above 495 nm.

### 2.5 Measurements of Mitochondrial and Cytosolic ROS

Mitochondrial ROS production was measured with fluorescent indicator 1 μM MitoTracker Red CM‐H2‐XRos (Invitrogen, USA) using a previously described protocol [24]. Cells were loaded with the probe for 10 min, which was kept in the solution during the experiments to avoid the intracellular accumulation of the oxidised probe. Fluorescent images were obtained by confocal microscopy (excitation 561 nm emission above 610 nm) and the rate of fluorescent increase was used as the rate of mitochondrial ROS production.

ROS were also imaged with dihydroethidium (Het) (Invitrogen, USA) using confocal microscopy. Confocal images were obtained with a Zeiss 900 LSM with an integrated META detection system. To avoid accumulation of oxidised products, HEt was not preincubated, but was present in solutions throughout the experiments. HEt was excited with the 588 laser and light emitted at 580–620 nm was measured.

### 2.6 GSH Measurements

Primary cultures of skeletal myotubes were incubated with 50 μm monochlorobimane (MCB) (Invitrogen, USA) for 40 min in HBSS prior to imaging. Then, cells were washed with HBSS and images of the fluorescence of the MCB‐GSH were acquired with excitation at 405 nm and emission at 435–485 nm using Zeiss 900 LSM [25].

### 2.7 MagFura‐2 Measurements

Mag‐Fura‐2 AM (Invitrogen by Thermo Fisher Scientific, USA) was used at 5 μM and loaded with 0.005% Pluronic P‐123 (Sigma‐Aldrich, USA) for 30 min at room temperature, followed by two washes with HBSS. Fluorescence measurements were obtained using an Olympus microscope with a CCD camera. Mag‐Fura was excited at 340 and 380 nm. Emitted fluorescence was reflected through a 515 nm long‐pass filter to a CCD camera. Images were analysed using MetaFluor imaging software and presented as the Mag‐Fura ratio (340 nm/380 nm).

### 2.8 Statistical Analysis

Statistical analysis and curve fitting were performed using Origin 2021 (Microcal Software Inc., Northampton, MA) software. Significance of the differences in independent experiments was determined by Kruskal–Wallis test with Dunn's post hoc test and in dependent experiments—by Wilcoxon test. Results are expressed as means ± standard error of the mean (SEM). All experiments were repeated at least three times.

## 3 Results

### 3.1 Mitochondrial Substrates Change ΔΨm of Myotubes

**FIGURE 1:**
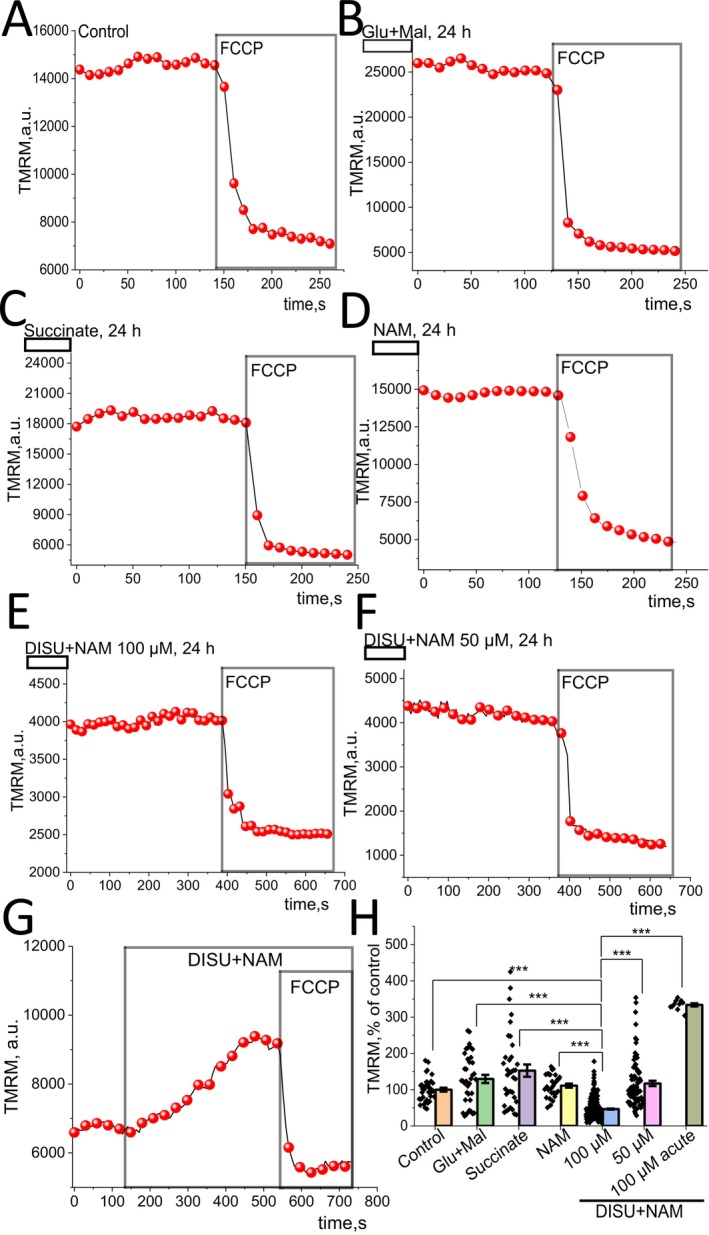
Effect of mitochondrial substrates and their combination on ΔΨm of myotubes. Representative traces of the TMRM fluorescence changes from the single myotubes (A–G). Incubation of skeletal myotubes with 10 mM/5 mM Glu + Mal (B), 10 mM Succinate (C), 10 mM NAM (D) changes ΔΨm (H). Combination of mitochondrial substrates 100 μM DISU+NAM (F) decreases mitochondrial potential, but at lower dose 50 μM (G) increases the mitochondrial membrane potential compared to control (H). Addition of 100 μM DISU+NAM leads to immediate significant increase of ΔΨm (H). 2 μM FCCP added for calibration of the signal and data in H represent the difference in TMRM fluorescence in myotubes before and after FCCP application. Data are represented as mean ± SEM. Kruskal–Wallis test with Dunn's post hoc test, *p < 0.05, **p < 0.01, ***p < 0.001.

The major indicator of mitochondrial ‘health’—mitochondrial membrane potential (ΔΨm) is maintained predominantly by the function of the electron transport chain (ETC) of mitochondria [26]. To estimate ΔΨm in primary myotubes, we used TMRM as a fluorescent indicator. Application of the mitochondrial uncoupler FCCP (2 μM) induces depolarization of mitochondria, and the difference between the basal TMRM level and the fluorescence after the addition of the protonophore was taken as ΔΨm in different cells (Figure 1A–H). The effect of mitochondrial substrates was studied after a 24‐h incubation. In skeletal myotubes, the 24‐h incubation with 5 mM glutamate (Glu) + 5 mM malate (Mal), 5 mM succinate or 5 mM nicotinamide didn't change ΔΨm (Figure 1A–D). However, the combination of the mitochondrial substrates which has been previously shown to be effective in neurons (DISU (DISU is a choline salt of succinic acid) + NAM) in a concentration 100 μM (but not 50 μM) reduced ΔΨm after a 24‐h incubation (Figure 1E,F,H) (46.4 ± 1.5% of control; *p* < 0.001). Yet, the acute application of 100 μM DISU+NAM induced a significant increase in ΔΨm of myotubes (333.8 ± 4.4% of control; *p* < 0.001) (Figure 1G,H).

### 3.2 Energy Metabolism Substrates Change Mitochondrial Redox Co‐Enzymes Pool and Redox Index

**FIGURE 2:**
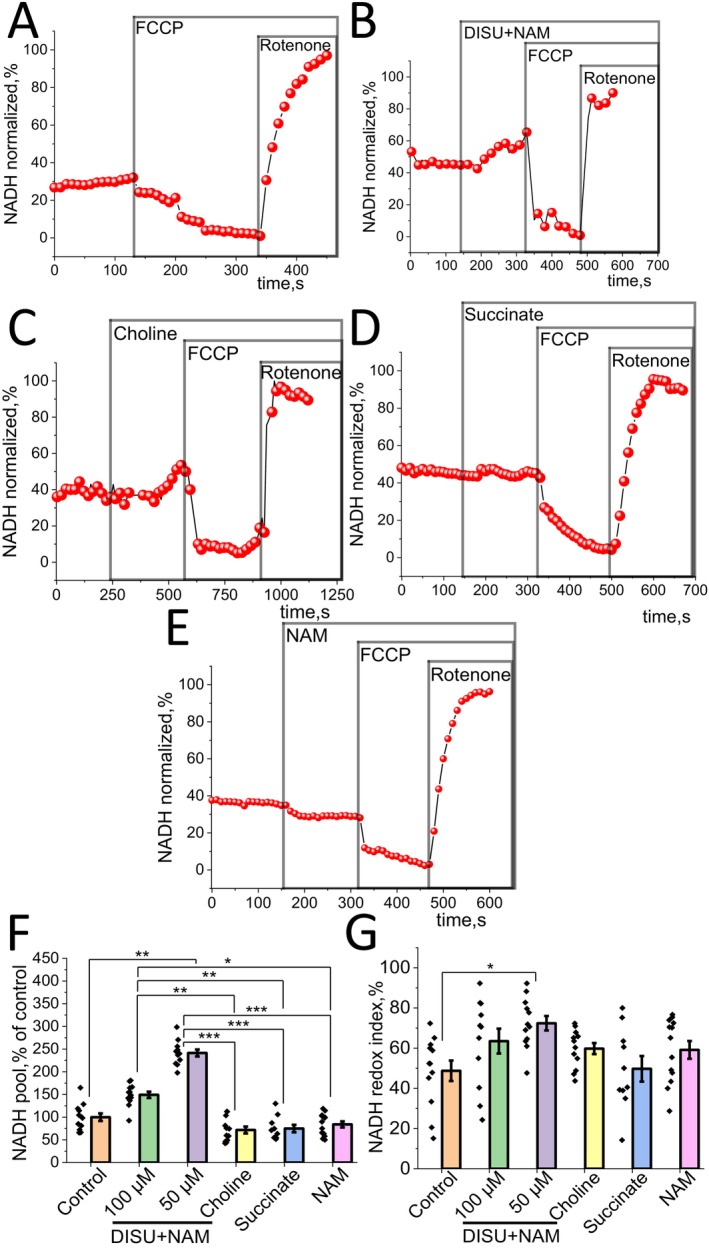
DISU + NAM increases mitochondrial NADH level in skeletal myotubes. (A–E) Representative NADH autofluorescence traces of myotubes after application of 1 μM FCCP and 5 μM Rotenone in: (A) control; (B) application of 50 μM DISU+NAM; (C) addition of 50 μM choline, (D) addition of 50 μM succinate, and (D) 50 μM NAM. Changes in mitochondrial NADH pool (F) or NADH redox index (G) of myotubes after application of various substrates. Data are presented as mean ± SEM. Kruskal–Wallis test with Dunn's post hoc test, *p < 0.05, **p < 0.01, ***p < 0.001.

One of the ways to evaluate mitochondrial metabolism in living cells and tissues is measuring the mitochondrial redox index [27, 28]. This could be done using measurement of the autofluorescence of NADH or FAD. To distinguish mitochondrial NADH from NADPH and non‐mitochondrial NADH, the application of protonophore FCCP (1 μM) was used for the activation of mitochondrial respiration that leads to minimal mitochondrial NADH level (taken as 0) while subsequent addition of 5 μM rotenone inhibits mitochondrial complex I and stops NADH consumption in mitochondria, and the values of fluorescence at this point could be taken as maximal NADH level in mitochondria (100%) (Figure 2A–E). This combination allows us to evaluate two redox characteristics: the relative NADH content (pool) in mitochondria and the redox index (the balance between NADH production and consumption in mitochondria of intact cells [Figure 2F,G]). Addition of DISU+NAM to myotubes at concentration 50 μM resulted in an increase in redox index (72.4% ± 3.6% in experimental group and 48.7% ± 5.1% for control cells, *p* < 0.05) and mitochondrial NADH pool compared to control (241.4% ± 7.7% of control, *p* < 0.05) (Figure 2A,B,F,G). 100 μM of the studied combination showed lesser and non‐significant increase of mitochondrial NADH parameters.

Experiments using the mitochondrial substrates—choline, succinate and NAM—led to a slight change of redox index (59.8% ± 2.6%, n. s.; 49.7% ± 6.4%, n. s.; 59.1% ± 4.4%, n. s.) and a statistically significant decrease of the NADH pool (71.7% ± 7.6%; 74.9% ± 8.2%; 84.0% ± 6.7%) compared to conditions where the complex of substances was used (Figure 2C–G). DISU+NAM was much more effective than the single applications of the substrates (Figure 2F,G).

Thus, the combination of mitochondrial substrates DISU+NAM caused an increase in NADH levels and NADH redox indices, in contrast to experiments where single mitochondrial substrates was applied. The data obtained indicate that the activation of energy metabolism in myotubes upon the addition of DISU+NAM has taken place.

**FIGURE 3:**
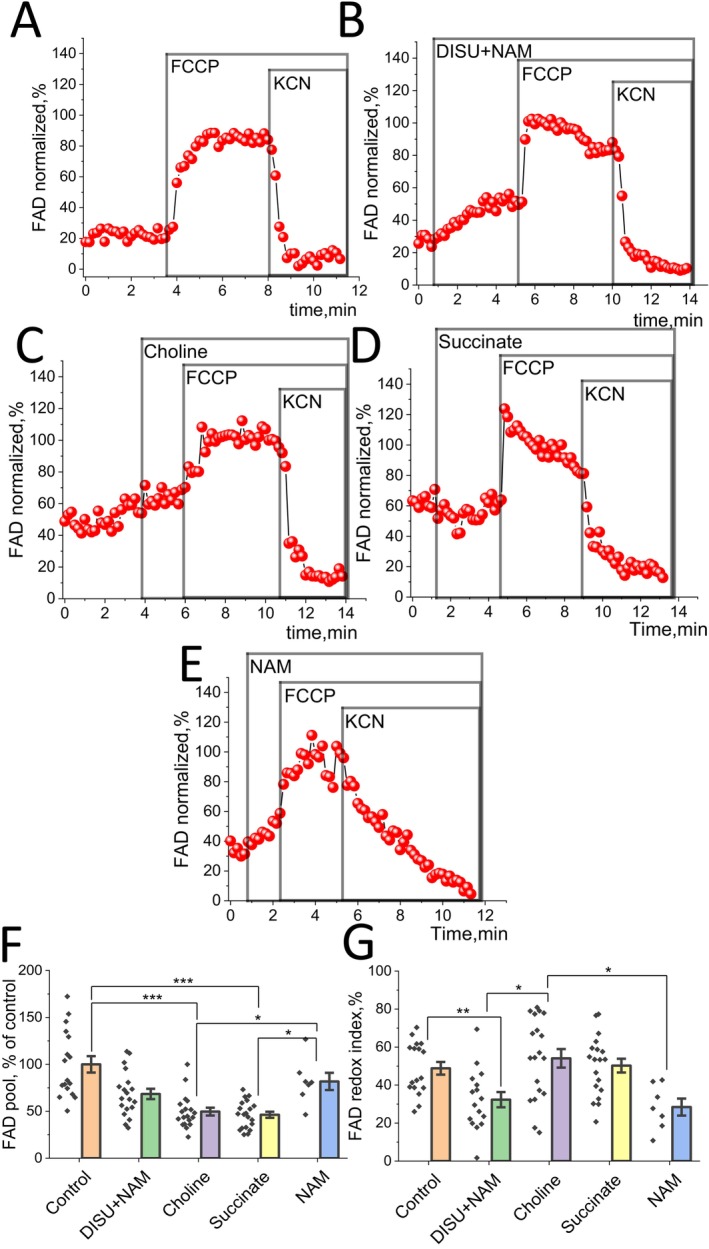
Effect of mitochondrial substrates on the FAD level and redox index in skeletal myotubes. (A–E) Representative traces of FAD autofluorescence of myotubes after application of 1 μM FCCP and 2 mM KCN: (A) control; (B) application of 50 μM DISU+NAM; (C) 50 μM choline; (D) 50 μM succinate; (E) 50 μM NAM. Changes in the FAD pool (F) or FAD redox index (G) of myotubes. Data are presented as mean ± SEM. Kruskal–Wallis test with Dunn's post hoc test, *p < 0.05, **p < 0.01, ***p < 0.001.

FADH is part of the flavoproteins playing an essential role as a donor of electrons and substrate of mitochondrial complex II. Thus, the level of FADH also demonstrates the functionality of mitochondria (Figure 3A). FAD++ is fluorescent and we used this autofluorescence to estimate the mitochondrial FAD pool and redox index. 1 μM FCCP and 2 mM KCN were used to maximally activate FADH consumption and block it in the same way as NADH, except for the fact that responses mirrored the effects of NADH because FAD is autofluorescent and FADH is not (Figure 3A).

The data obtained allowed us to evaluate the bioenergetic state of cells by the content of the coenzyme FAD in myotubes. A statistically non‐significant decrease in the levels of FAD (68.5% ± 5.5% of control) and more considerable—in the redox index (32.3% ± 4.0%, *p* < 0.01) was observed upon addition of DISU+NAM in comparison to the control experiment (Figure 3A,B).

Addition of mitochondrial substrates choline and succinate showed a statistically significant decrease in the FAD pool (49.7% ± 4.2% of control, *p* < 0.001; 46.3% ± 3.2% of control, *p* < 0.001). At the same time, the redox index (54.1% ± 4.9%; 50.3% ± 3.6%) did not change compared to the control but was increased for choline compared to the addition of the substrates (Figure 3A–F). However, NAM, which is a precursor of complex I coenzyme, leads to a statistically significant decrease in the redox index (28.4% ± 4.5%, *p* < 0.05) compared to DISU + NAM, whereas the FAD level (81.8% ± 9.2%) remains at a level comparable to the control as well as the combination of substrates (Figure 3E).

Thus, mitochondrial substrates in the form of DISU+NAM slightly reduce the level of FAD and lead to an increase of relative FADH2 level, showing a more significant role of mitochondrial complex II ETC function (Figure 3F,G).

### 3.3 DISU + NAM Increases ATP Energy Capacity

**FIGURE 4:**
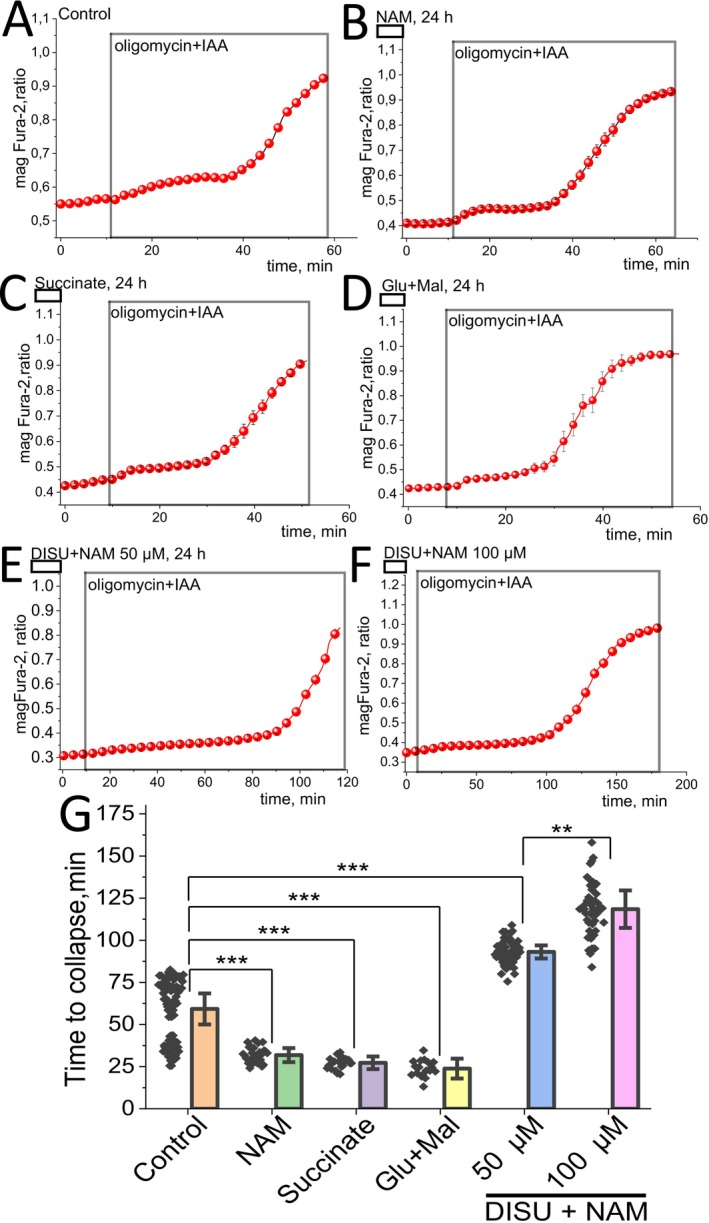
Incubation of myotubes with substrates of mitochondrial metabolism changes energy capacity in these cells. Representative traces of the changes in the magFura‐2 ratio of primary myotubes after application of 2 μg/mL oligomycin +20 μM IAA in control myotubes (A) and cells after 24‐h incubation with 10 mM NAM (B),10 mM Succinate (C), 10 mM/5 mM Glu + Mal (D), 50 μM DISU+NAM (E) and 100 μM DISU+NAM (F). (G) Time from application of oligomycin and IAA to collapse of myotubes. Data are represented as mean ± SE. Kruskal–Wallis test with Dunn's post hoc test, *p < 0.05, **p < 0.01, ***p < 0.001.

The energy capacity of the cell is defined as the time between cessation of ATP production and the time of energetic collapse due to a total ATP depletion (Figure 4A) and inability to maintain calcium homeostasis [29, 30]. Live‐cell imaging of the fluorescent probe MagFura‐2 AM was used to assess the energy capacity of primary myotubes. ATP is stored as a magnesium salt in the cells. Upon hydrolysis of ATP, Mg^2+^ is released from the Mg^2+^‐ATP complex; therefore, measurement of the changes in the cellular free magnesium using the Mg^2+^ sensitive fluorescent probe MagFura‐2 can be used as an indirect reporter for the rate of ATP consumption [31]. Application of inhibitors of glycolysis and/or OXPHOS blocks ATP production in cells, which leads to utilisation of the available ATP in the cell and a subsequent Mg^2+^ release. In addition to binding to Mg^2+^, the MagFura‐2 probe is also a low‐affinity Ca^2+^ indicator that can help detect high cytosolic calcium rise in the time of cell lysis, that is, the energetic collapse due to a total cellular ATP depletion and the inability of the cell to maintain Ca^2+^ homeostasis. This enables the estimation of the cellular energy capacity. In our experiments, a 24‐h incubation of the myotubes with mitochondrial substrates: NAM, succinate, Glu + Mal (Figure 4B–D) surprisingly decreased the energy capacity of myotubes (31.9 ± 0.8 min, 27.2 ± 0.8 min, 23.9 ± 1.2 min, *p* < 0.001 for al experiments) compared to control (Figure 4A) (59.2 ± 1.8 min). However, a 24‐h incubation of myotubes with the complex of mitochondrial substrates DISU+NAM in two concentrations (50 μM and 100 μM) increased the time to collapse in myotubes (93.1 ± 0.8 min, 118.5 ± 2.2 min, *p* < 0.001 for al experiments) (Figure 4E–G), strongly suggesting the increase of energy capacity of these cells compared to untreated cells. It should be noted that the effect of 100 μM substrates combination is 1.3 times higher compared to 50 μM (*p* < 0.01). It should be noted that the rate of ATP consumption changed during the experiment (Figure 4A–F) that also could be dependent on the calcium activity. Thus, providing the cells with a combination of DISU+NAM for energy metabolism increased ATP levels and enhanced the energy capacity of myotubes.

### 3.4 DISU+NAM Increases Mitochondrial ROS Production

**FIGURE 5:**
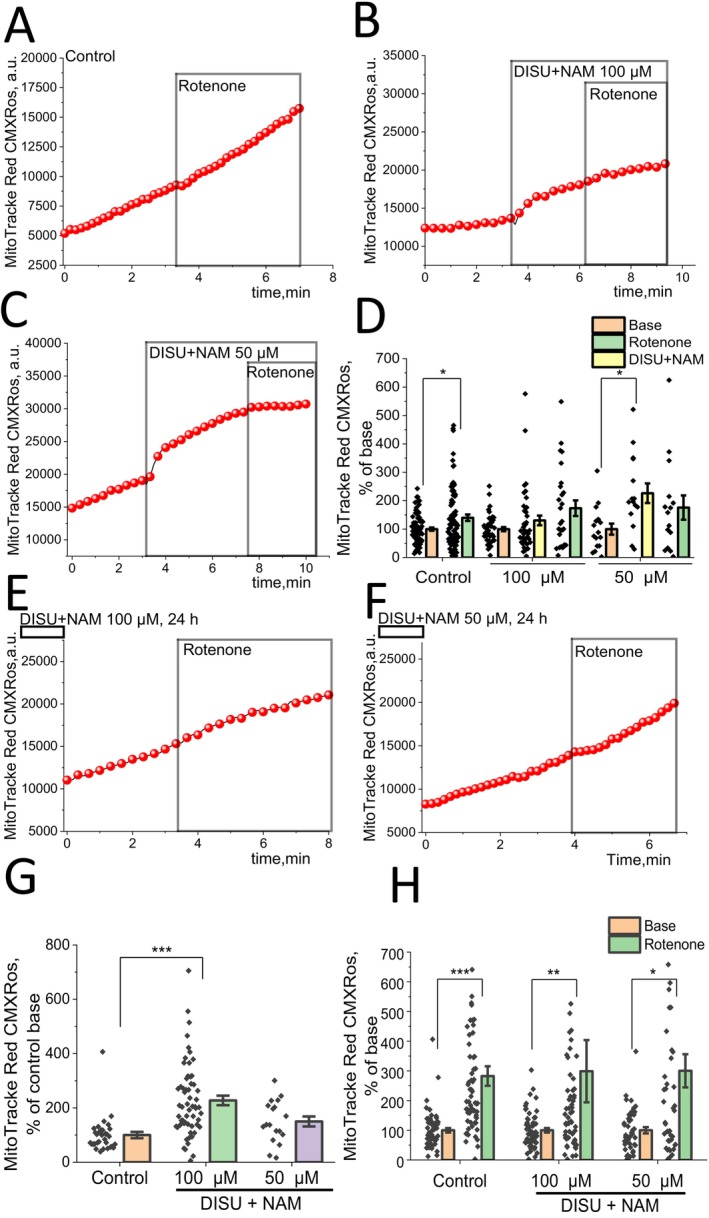
DISU + NAM increases mitochondrial ROS production in skeletal myotubes. Representative traces of experiments with average signal of MitoTracker Red CMXRos fluorescence, in control cells (A) and cells after addition DISU+NAM in two concentrations 100 μM (B) and 50 μM (C). Rotenone (5 μM) added in the end of experiments increases mitochondrial ROS production in experiments with addition of DISU+NAM and in control experiment (D). Prolonged 24‐h incubation with DISU+NAM (100 μM and 50 μM) (E, F) also increases the rate of mitochondrial ROS production and rotenone‐induced ROS production (H). Data are presented as mean ± SEM. Kruskal–Wallis test with Dunn's post hoc test (G) and Wilcoxon test (D, H), *p < 0.05, **p < 0.01, ***p < 0.001.

Production of ROS in mitochondria is dependent on the mitochondrial membrane potential and the metabolic state of the cells [32]. Considering the profound effects of the mitochondrial substrates on ΔΨm of mitochondria in myotubes, we suggested that it may also influence mitochondrial ROS production. In control myocytes, application of the inhibitor of mitochondrial complex I, rotenone (5 μM) induced an increase in the rate of mitochondrial ROS production (Figure 5A,D). Addition of DISU+NAM to myotubes during the experiment results in an increase in the rate of ROS production in mitochondria of myotubes at 100 μM concentration (130.5 ± 17.1% of basal level; n.s.; Figure 5B,D) and an even higher increase in ROS production at 50 μM DISU+NAM (226.4% ± 34.5% of basal rate; *p* < 0.05; Figure 5C,D). Subsequent application of rotenone to these cells induced a further increase in the rate of ROS production at 100 μM DISU+NAM (173.7% ± 27.5% of basal level n.s.) and a decrease at 50 μM of DISU+NAM (175.0% ± 42.7% of basal level, n.s.) (Figure 5A–D).

A 24‐h incubation of myotubes with DISU+NAM also leads to an increase in the rate of mitochondrial ROS production at 100 μM (227.5% ± 17.6% of untreated control; *p* < 0.05) or 50 μM (150.1% ± 18.4% of control, n.s.) (Figure 5E–G). Prolonged incubation of the cells with DISU+NAM did not change the rotenone‐induced ROS production in mitochondria at concentrations of 100 μM (299.0% ± 104.9% of basal level; *p* < 0.01) and 50 μM (300.3% ± 55.9% of basal level; *p* < 0.05) compared to control (282.6% ± 32.8% of basal level; *p* < 0.001) (Figure 5E–H).

### 3.5 Effect of DISU+NAM on the Rate of Cytosolic ROS Production

Mitochondria could produce ROS not only into the matrix of this organelle but also outside. Additionally, mitochondrial substrates may have an effect on ROS‐producing enzymes in the cytosol [33]. To assess the rate of ROS production, the fluorescent indicator dihydroethidium (HEt) was used.

**FIGURE 6:**
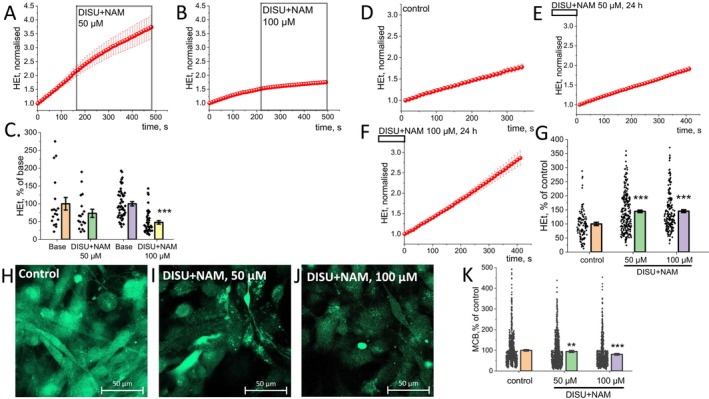
Effect of DISU+NAM on redox balance of skeletal myotubes. (A, B) Representative traces of HEt fluorescence intensity increases before and after DISU+NAM addition (50 and 100 μM). Quantification of changes in HEt oxidation rate (C) (data are shown as a percentage of the basal level of HEt fluorescence increase rate before DISU+NAM addition). (D–F) Representative trace of HEt fluorescence intensity increase in control myotubes and after 24‐h incubation with 50 and 100 μM of DISU+NAM. (G) Quantification of changes in HEt oxidation rate after 24‐h incubation with different concentrations of DISU+NAM (data are shown as a percentage of control). (H–J) Representative confocal images of myotubes loaded with MCB. Scale bar 50 μm. (K) Quantification of GSH‐MCB fluorescence intensity in myotubes. Incubation with DISU+NAM for 24 h decreases the level of glutathione. Data are presented as the mean ± SEM. Kruskal–Wallis test with Dunn's post hoc test (G, K) and Wilcoxon test (C), *p < 0.05, **p < 0.01, ***p < 0.001.

To determine whether ROS production could be activated, the DISU+NAM complex was added at concentrations of 50 and 100 μM after recording a baseline of HEt fluorescence (Figure 6A,B). The degree of activation of ROS production was calculated as a percentage of the baseline fluorescence level (Figure 6C). Application of DISU+NAM in both cases leads to a statistically significant decrease in the HEt fluorescence rate at a concentration of 50 μM (73.2% ± 11.4% of basal level, *p* < 0.01) and 100 μM (47.7% ± 4.8% of basal level, *p* < 0.001).

However, a 24‐h incubation of myotubes with DISU+NAM resulted in the opposite effect (Figure 6D–F). There was an increase in the rate of ROS production with DISU+NAM at both 50 μM (144.5% ± 4.5% of control, *p* < 0.001) and 100 μM (145.3% ± 5.2% of control, *p* < 0.001) concentrations compared to control cells (Figure 6G).

### 3.6 A 24‐h Incubation of Myotubes With DISU+NAM Results in Decreased Levels of Reduced Glutathione

Glutathione is one of the most important components of the cellular antioxidant system. The content of the reduced form of GSH in myotubes was determined using MCB, which acquires fluorescence ability upon interaction with GSH (Figure 6H–J). Cells were incubated with DISU+NAM for 24 h, then GSH levels were measured. The incubation of the myotubes with DISU+NAM for 24 h decreased the level of GSH: 50 μM (94.5% ± 3.2% of control, *p* < 0.001) as well as at a concentration of 100 μM (80.6% ± 2.6% of control, *p* < 0.001) (Figure 6K). Thus, the incubation of the skeletal myotubes with energy substrates induces ROS production in mitochondria and cytosol with statistically significant but relatively minor effects on endogenous antioxidant GSH levels.

### 3.7 DISU+NAM Increases the Diameter of Muscle Fibres

Systematic and intensive work of a muscle promotes an increase in muscle tissue mass. This phenomenon is called muscle hypertrophy. The basis of hypertrophy is an increase in the mass of the cytoplasm of muscle fibres and the number of myofibrils contained in them, which leads to an increase in the diameter of each fibre. At the same time, the synthesis of nucleic acids and proteins is activated in the muscle and the content of substances delivering energy used in muscle contraction—adenosine triphosphate and creatine phosphate, as well as glycogen—increases. As a result, the strength and speed of contraction of the hypertrophied muscle increase. To assess the possibility of mitochondrial substrates to decrease muscle atrophy degree, an aged model of sarcopenia was used [34].

**FIGURE 7:**
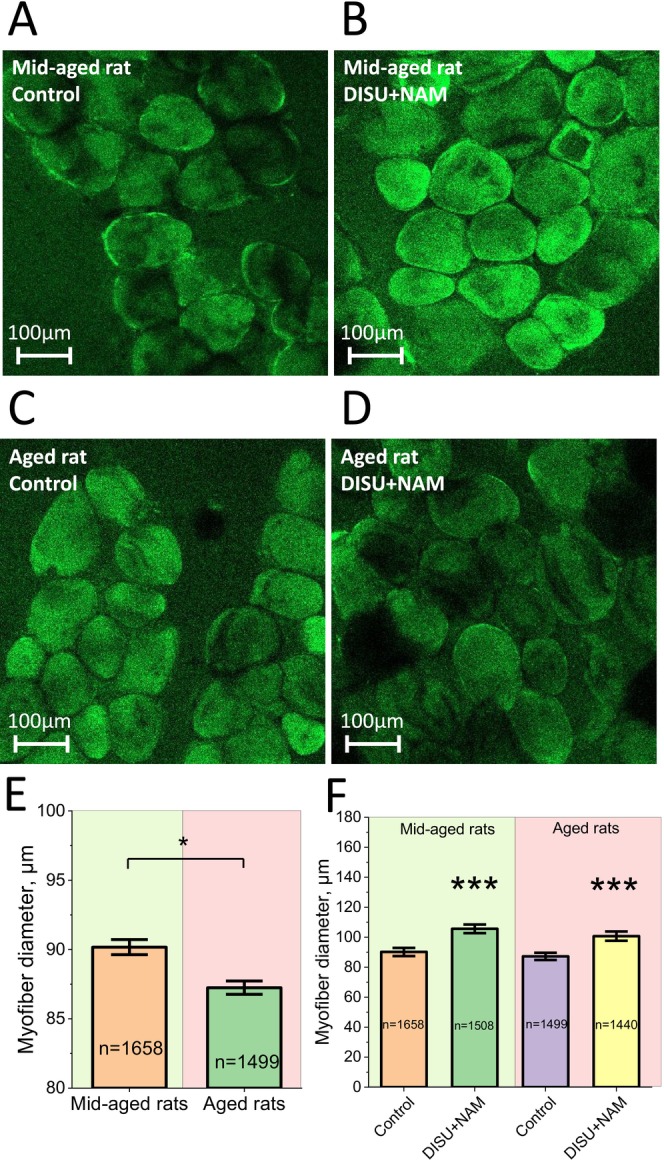
The DISU + NAM combination increases the diameter of muscle fibres in aged and young animals. The diameter of muscle fibres from striated muscle tissues was analysed by confocal microscopy with fluorescent sensor Fluo‐4 (5 μM) and 0.005% Pluronic acid. Example of confocal image of a tissue slice from a group of young animals intact (A) and treated with DISU+NAM for 7 days (B). Example of confocal image of a tissue slice from a group of old animals intact (C) and treated with DISU+NAM for 7 days (D). Scale bar 100 μm. (E) Statistically significant difference between the diameter of muscle fibres in the two groups. (F) Change in muscle fibre diameter during treatment with DISU+NAM in drinking water ad libitum at a concentration of 100 μM for 7 days. The diameter of muscle fibres increased statistically significantly in the groups of old and young animals. Data are presented as mean ± SEM. Kruskal–Wallis test with Dunn's post hoc test, *p < 0.05, ***p < 0.001.

Animals were treated with DISU+NAM through the drinking water ad libitum at a concentration of 100 μM for 7 days. To analyse the diameter of muscle fibres, acute sections of transverse striated muscles of hind legs were incubated in saline solution in the presence of fluorescent probe Fluo‐4 AM for 30 min. We observed that in the animals in the aged group (Figure 7C), the diameter of muscle fibres decreased compared to the younger group (Figure 7A). Muscle mass decreases with age, resulting in sarcopenia (Figure 7E). On average, the diameter of muscle fibres in 1‐year‐old (mid‐aged) rats was 90.2 ± 0.5 μm, which is higher than that in the group of aged (1.5‐year‐old) animals 87.2 ± 0.5 μm (*p* < 0.05). In the groups receiving the mixture of substrates, an increase in muscle fibre diameter was observed compared to the control group to 105.6 ± 0.6 μm and 100.7 ± 0.6 μm for the young (Figure 7B) and aged groups (Figure 7D), respectively (*p* < 0.001 for all experiments).

## 4 Discussion

Sarcopenia is defined as ‘a progressive and generalised skeletal muscle disorder involving the accelerated loss of muscle mass and function that is associated with increased adverse outcomes, including falls, functional decline, frailty and mortality [17]. Although sarcopenia is recognised as a disease with an International Classification of Diseases code [35], there is a lack of consensus with regard to its clinical identification [36, 37]. Its multifactorial pathogenesis includes enhanced expression of muscle growth inhibitors and oxidative stress, neuromuscular junction dysfunction, impaired function of muscle stem cells (MuSCs), reduced mitochondrial biogenesis and function, diminished muscle protein synthesis, activation of catabolic pathways and development of insulin resistance. These attributes lead to muscle fibre reorganisation, myofibril degeneration, and myocyte death, as described in detail in a series of reviews [38, 39, 40, 41, 42]. Here, we show that in only 7 days support supplementation of the animals with DISU+NAM fibre diameter in both mid‐age and aged rats increases, abolishing age‐related sarcopenia. It should be noted that although DISU+NAM increased the diameter of myotubes of both ages, the most important is the finding that age‐related sarcopenia could be induced by the lack of energy and could be partially recovered by energy substrates. It is also interesting that our results also suggest that the size of muscle fibre in young rats could also be diminished by lack of energy (Figure 7).

Interestingly, the combination of substrates for energy metabolism (DISU+NAM) is much more effective compared to the use of the single components or other substrates. Thus, this could be explained by the lower permeability of the plasma membranes for succinate. Further, muscle cells have a very low capacity for uptake of not only succinate but also for uptake of glutamate [43]. Recently, we have shown that the application of various substrates to the brain cells induced fast activation of mitochondrial energy metabolism and that the combination of the substrates—such as DISU‐ accelerated these effects, suggesting an effect on cell permeability [23].

It should be noted that the influence of DISU+NAM on most of the assessed parameters is concentration dependent. A more pronounced effect of 50 μM of the substrates mixture was revealed during acute application (excluding ΔΨm measurements) while after 24‐h‐incubation, 100 μM of DISU+NAM was more stimulating, especially in the case of time to collapse (Figure 4G) indicating the ATP energy capacity. A smaller effect of 100 μM DISU+NAM on mitochondrial membrane potential and NADH pool may be explained by the activation of mitochondrial respiration and ATP consumption (i.e., confirmed by other experiments) but also by a possible non‐mitochondrial effects such as short‐term acidification of the cytosol with high concentrations of mitochondrial substrates [44]. Comparison of the data obtained with the results of similar research with neurons [23] shows the higher sensitivity of myotubes to DISU+NAM supplementation due to the high metabolic plasticity of muscle cells [45]. Although in our experiments, the enhancement of energy metabolism led to an increase in ROS production in the mitochondria and cytosol of myotubes, it may have a regulatory effect on energy metabolism and myotube activity [46]. Additionally, activation of NADPH oxidase in the post‐exercise period was shown to also act as a redox activator of energy metabolism [47], which could also be the reason for the relatively lower GSH in the myotubes treated with DISU+NAM.

## Author Contributions

**Andrey Y. Vinokurov:** conceptualization (equal), resources (equal), writing – original draft (equal), writing – review and editing (equal). **Pavel A. Bazhenov:** data curation (equal), formal analysis (equal), investigation (equal), writing – original draft (equal). **Marina Y. Pogonyalova:** data curation (equal), formal analysis (equal), investigation (equal), writing – original draft (equal). **Evgenia S. Seryogina:** data curation (equal), formal analysis (equal), investigation (equal), writing – original draft (equal). **Ekaterina A. Vetrova:** data curation (equal), formal analysis (equal), investigation (equal), writing – original draft (equal). **Larisa Andreeva:** conceptualization (equal), supervision (equal), writing – review and editing (equal). **Andrey Y. Abramov:** conceptualization (equal), supervision (equal), validation (equal), writing – original draft (equal), writing – review and editing (equal). **Plamena R. Angelova:** conceptualization (equal), methodology (equal), resources (equal), supervision (equal), validation (equal), writing – review and editing (equal).

## Ethics Statement

This study was approved by the Institutional ethical committee of Orel State University (No. 18 dated 21 February 2020).

## Conflicts of Interest

L.A. is an employee of Mitocholine Ltd. All other authors declare no conflicts of interest.

## Data Availability

Data will be made available on reasonable request.
